# Supplementary material for: Fragmentation of hunting bullets observed with synchrotron radiation: Lighting up the source of a lesser-known lead exposure pathway
Source: PLoS One. 2022 Aug 24;17(8):e0271987. doi: 10.1371/journal.pone.0271987 (PMC9401160; doi:10.1371/journal.pone.0271987)
Supplement: S5 File — (DOCX) [file pone.0271987.s005.docx]

**Supporting information**

Fragmentation of hunting bullets observed with synchrotron radiation: Lighting up the source of a lesser-known lead exposure pathway

Adam F. G. Leontowich^1^*, Arash Panahifar^1,2^, Ryan Ostrowski^3^

1. Canadian Light Source Inc., Saskatoon, Saskatchewan, Canada

2. University of Saskatchewan College of Medicine, Department of Medical Imaging, Saskatoon, Saskatchewan, Canada

3. Royal University Hospital, Saskatoon, Saskatchewan, Canada

* Corresponding author

Email: adam.leontowich@lightsource.ca (AL)

**Procedure to create K edge subtracted (KES) images**

The maximum field of view of the BMIT-ID beamline could not capture a whole sample in one exposure, so each monochromatic image is a composite of 40 exposures (20 V × 2 H) stitched together using a routine [1] in the program ImageJ. For each sub-region and energy, three images were collected: 1) The raw image, *I* (beam on, sample in the beam), 2) a flat field image, *I*_0_ (beam on, no sample in the beam), and 3) a dark image, *D* (beam off). All of the data required to produce a KES image could be collected in 20 minutes.

Transmission images of each sub-region were created using the equation (Equation 1),

$T=\frac{I-D}{I_{0}-D}$ (1)

The 40 sub-regions were then spatially aligned (Fig 4a and 4b). The resulting images were converted from transmission to absorption by taking the natural logarithm and multiplying by -1, and then the KES image was obtained by subtracting the 87 keV image from the 89 keV image. The absorption scale was converted to micrometer thickness lead scale using the measured absorption of the lead foil internal standard, which was within 10% of the pre-edge subtracted literature value for 165 µm lead at 89 keV [2].

A few fragments, which were all among the largest observed, lost significant contrast in the KES image. This observation might suggest these fragments could be a mixture of copper and lead. However, further analysis of the images revealed the largest fragments had very low transmission of <1% (Absorption >4.6) even at 87 keV. As transmission decreases, the signal to noise ratio increases, reducing the accuracy and introducing an upper limit of linearity for quantification. The X-ray transmission of 150 mm of ballistic gelatin was 9.0% (Absorption = 2.4), and the transmission of the 165 µm lead foil within the gel at 89 keV was 3.7%, (Absorption = 3.3). Fragments with thickness >200 µm are estimated to be above the limit of linearity for quantification in this example, though they can still be qualitatively categorized as lead.

To check for the presence of copper in the large highly absorbing fragments, 12 large bullet fragments were targeted and cut out of the lead-core bullet samples. These extracted fragments were measured with calipers and all had at least one dimension greater than 1 mm. All appeared to be lead under an optical microscope, with no trace of copper color. These extracted fragments and a lead-core bullet pulled from an unfired cartridge underwent X-ray fluorescence spectroscopy measurement at the IDEAS beamline at the Canadian Light Source. The Ge (220) double crystal monochromator was set to an incident energy of 13.75 keV, and X-ray fluorescence was collected using a Hitachi Vortex ME4 silicon drift detector. The signal from the jacket of the lead-core bullet was dominated by copper emission, with a significant contribution from zinc, as expected. There was no significant lead signal from the jacket of the lead-core bullet. In contrast, the X-ray fluorescence signal from all 12 extracted fragments was dominated by lead emission. There was no contribution from copper or zinc, confirming that the largest lead fragments experienced some absorption saturation in the KES image.

**References**

[1] Preibisch S, Saalfeld S, Tomancak P. Globally optimal stitching of tiled 3D microscopic image acquistions. Bioinformatics 2009;25(11):1463-1465.

[2] Hubbell JH, Seltzer SM. Tables of x-ray mass attenuation coefficients and mass energy-absorption coefficients. National Institute of Standards and Technology: Gaithersburg, USA, 2004. DOI: https://dx.doi.org/10.18434/T4D01F
