# Supplementary material for: Fragmentation of hunting bullets observed with synchrotron radiation: Lighting up the source of a lesser-known lead exposure pathway
Source: PLoS One. 2022 Aug 24;17(8):e0271987. doi: 10.1371/journal.pone.0271987 (PMC9401160; doi:10.1371/journal.pone.0271987)
Supplement: Attachment — Submitted filename: Response to Reviewers.docx [file pone.0271987.s006.docx]

June 14, 2022

Response to reviewer’s comments

Manuscript reference: PONE-D-22-05415

Leontowich AFG *et al*., “Fragmentation of hunting bullets observed with synchrotron radiation: Lighting up the source of a lesser-known lead exposure pathway”, Submitted to PLOS ONE.

We appreciate the time and effort the reviewers and editor have put toward considering our manuscript for publication, and we thank the reviewers for their clear and detailed constructive feedback and comments. We have carefully considered all of the comments and have prepared a revised manuscript. Our response for each comment is noted below in blue.

Reviewer #1: The authors used synchrotron K edge subtraction imaging to investigate the fragmentation of a traditional lead-core bullet after impact on ballistic gelatin and compared it with the fragmentation of a copper bullet. The synchrotron K edge subtraction imaging method was applied the first time on this subject and allowed to image specifically the lead fragments. A statistical analysis of the size distribution of the lead particles down to particle sizes of a few micrometer showed for the first time that the vast majority of particles is smaller than detectable by visual inspection.

The study is very clearly and carefully written, technically sound and the topic is of relevance for human health and environment.

However, the study has some limitations in my view, which the authors should comment on:

1. The study is based only on four impacts: two with a lead-core bullet (with and without bone), and two with a copper bullet (with and without bone).

a. It is not clear whether the results of this particular lead-core bullet can be generalized to all or other popular types of lead-core bullets.

b. It is not clear whether this single impact is representative for this bullet type.

c. It is not clear whether the fragmentation is the same for ballistic gelatin, which is used in the study, and for a real big game animal.

This is a good question. We have greatly expanded on our partial answer (included below in italics), and have added the following to the manuscript:

Rifles are manufactured in dozens of calibers, and there are dozens of ammunition manufacturers worldwide, with each producing several types of hunting bullets. Shot distances can range from 10 m to over 500 m. There are several species of big game animals, and each species has several tissue types where the bullet impact could happen. These variables lead to thousands of possible impact outcomes. Fortunately, the literature contains hundreds of data points on hunting bullet impacts, including those from all major ammunition manufacturers, in all common calibers, at a range of distances, in many types of animal tissue and tissue simulants including ballistic gelatin. Three key and generalizable traits have emerged in the literature,

1) Traditional lead-core hunting bullets produce many metallic fragments on impact, also referred to as a “lead snowstorm”.
2) All-copper hunting bullets produce few to no metallic fragments on impact.
3) The size of the wound channels has been characterized for both bullet types, at typical hunting distances in a range of tissues and tissue simulants.

Although our study is based upon 4 impacts, due to the limited amount of synchrotron beamtime available, *our observations of fragmentation and wound channel dimensions are in agreement with previous studies [11-13,16,23,34]. They indicate that* the impacts are representative for the bullet type, and that *ballistic gelatin is a fine tissue substitute [1,35].*

2. The synchrotron K edge subtraction imaging method is standard at synchrotrons. The novelty of this study lies therefore exclusively in its application. Would the method also work with similar sensitivity on real big game animals? Please comment on expected limitations.

In brief, we have since performed follow-up studies of hunting bullet wounds in big game animal tissue and can confirm that we see the equivalent results in animal tissue and ballistic gelatin. The animal tissue results will be the subject of a new manuscript.

The technical limitations of the technique have been included in the supplementary section. It comes down to having enough transmission through the sample and the fragments to get an accurate absorption measurement. At 88 keV around the lead K edge, ~150 mm thickness of tissue is going to be about the limit. Also, fragments with thickness >200 µm are too thick to get great statistics, though they can still be qualitatively identified as lead. We also made this note in the manuscript, which summarizes the paragraph into one sentence,

“Absorption saturation would also be an issue as X-ray attenuation lengths for all materials decrease with decreasing photon energy [40], so samples may need to be thinned.”

Practically, synchrotron beamtime access is going to be the bottleneck. Our answer for question 1 which we have added to the manuscript, now briefly mentions limited synchrotron access. The scans take about 20 minutes per block, but that does not include many hours of beamline and sample alignment, sample stage and photon energy calibrations. Our samples were about as big as the current motorized sample stages at the BMIT facility can handle. Whole deer are likely not possible, however a whole small animal or whole game bird is possible.

3. What is the market share of lead-core bullets compared to nonlead-core bullets today? Are lead-core bullets still relevant?

We reached out by email to Federal Ammunition, the manufacturer of the ammunition used in the manuscript, and received a response in less than 24 hours,

The question:

Hello,

Last season I tried Federal Non-Typical all-copper bullets in 308W for my white-tail deer hunt and was very impressed with the performance. I was wondering, could you say approximately what is the market share of all-copper rifle ammo to standard lead-core ammo? I would imagine it's growing but probably remains low, as most people shoot +100 lead rounds at targets for every 1 copper round at an actual deer. Thank you,

Adam

The response:

Adam

California is the only state that has banned lead bullets for hunting, other states are pushing it but until that happens in more states the ratio for all copper bullets is still very small compared to the lead core bullets. Complete guess would be less than 3% of the rounds sold, but don’t have any specific data.

Thank You

Federal Ammunition

Tech Services

I can also provide my own opinion as hunter in my region of Canada: Lead-core bullets are definitely still relevant. Most white-tail deer hunters don’t realize there is an issue or that there are other options. To be honest I didn’t know until I started this study and I have hunted for 10 years.

4. In line 346 ff you reason about the maximum extent that fragments may be distributed in a harvested big game animal. I suggest to use your data to compile a size dependent radial distribution of the fragments (of cause you only have the measurement of the projection of the distribution, estimate the effect).

Fragment composition, size, amount, and distribution all affect toxicity. There are many examples in the literature that focus heavily on the number of fragments, and their radial distribution. Our work is really focused on fragment composition and size, therefore we do not feel it is necessary to include an additional figure to the manuscript. If the reader is interested in that analysis, the radiographs and KES image figures include scale bars, and all the raw data will be included in an online repository as well.

In conclusion, the publication of the study is recommended. The comments should be considered. In the best case it helps to expulse lead-core bullets worldwide. Whether PLoS ONE is the appropriate journal is to be assessed by the editor.

Reviewer #2: In the paper, KES imaging is used to identify lead bullet fragments in animal remains among other metal (copper) and bone fragments. High resolution capabilities of the technique is also evaluated. A comparison with the widely diffused medical radiography imaging shows the advantages of the synchrotron KES imaging.

Material and methods are well discussed and results fully reproducible. Bibliography is up-to-date and pertinent. Text is clear, logical and well written.

Just a few remarks: in eq.1 line 206, “t” where (line 208) “t is thickness (cm)” makes no sense. It should be lambda, i.e. mass thickness in g/cm2.

We thank the reviewer for catching our error. We missed an extra term *ρ* in equation 1. We have changed equation 1 to,

I=I_0_e^-(*µ*/*ρ*)***ρ****t*

We double checked all our work related to this equation and confirm the analysis was done with the extra term in place. It was simply a typo, and no changes needed to be made to the analysis in the manuscript. We confirm the revised equation is correct. It comes directly from reference [40], https://physics.nist.gov/PhysRefData/XrayMassCoef/chap2.html

I would move line from 266 to 292 in the Introduction (it’s basically bibliography) The same for lines from 324 to 333 and from 338 to 345.

We feel it would be difficult to do this and maintain the current flow of the manuscript. Both reviewers have made comments approving of our writing style. Therefore we prefer to leave these sections in their current locations.

ImageJ software needs to be described in the Methods.

We have added in the following line to the Materials and Methods section, and added another reference,

Image processing was performed with the program ImageJ.

Schneider CA, Rasband WS, Eliceiri KW. NIH Image to ImageJ: 25 years of image analysis. Nat. Meth. 2012;9:671-675.

Is the last paragraph “Alternatives to traditional rifle ammunition” really required in a research paper like this? I feel it’s redundant.

This 350 word section returns the reader to the motivation for this work: Encouraging hunters to choose non-toxic rifle ammunition. We agree with the reviewer’s point that alternatives to traditional rifle ammunition have been around for decades now and are not novel. There are numerous literature examples on exactly this topic, which we also cite in this section. Researchers in the area are well aware that alternatives exist.

However, in my opinion most Canadian white-tail deer hunters don’t realize there is an issue or that there are other options. To be honest I didn’t know until I started this study and I have hunted for 10 years. With an open-access journal like PLOS ONE, everyone will be able to access this work and possibly learn for the first time about the issue and the alternatives. We do include here an up to date summary of the current state of Canadian laws and messaging from the provinces, which has not been recently published in the literature to our knowledge.
